# Impact of out-of-pocket expenses on children with cancer in Tanzania: A mixed-methods economic study

**DOI:** 10.1371/journal.pone.0326755

**Published:** 2025-06-26

**Authors:** Emily R. Smith, Pamela Espinoza, Happiness D. Kajoka, Henry E. Rice, Madeline Metcalf, Anna Tupetz, Cesia Cotache-Condor, Blandina T. Mmbaga, Catherine Staton, Esther Majaliwa

**Affiliations:** 1 Department of Emergency Medicine, Duke University Medical Center, Durham, North Carolina, United States of America; 2 Duke Global Health Institute, Duke University, Durham, North Carolina, United States of America; 3 Kilimanjaro Christian Medical University College, Moshi, Tanzania; 4 Division of Pediatric Surgery, Department of Surgery, Duke University Medical Center, Durham, North Carolina, United States of America; 5 Kilimanjaro Clinical Research Institute, Kilimanjaro Christian Medical Centre, Moshi, Tanzania; 6 Pediatric Hematology and Oncology Services, Kilimanjaro Christian Medical Centre, Moshi, Tanzania; University of Nigeria, Enugu Campus, NIGERIA

## Abstract

**Background:**

For children with cancer in low- and middle-income countries, medical and non-medical expenses are often paid through out-of-pocket (OOP) expenditures, which pose significant barriers to timely care. Our study aims to estimate the impact of OOP expenditures for cancer care for children in Tanzania through a mixed-methods approach.

**Methods:**

We used an explanatory mixed-method design to evaluate the impact of OOP expenditures for children receiving cancer care at the Kilimanjaro Christian Medical Center in Tanzania based on the Three Delays Framework. Quantitative data were collected to measure OOP expenditures and to assess the risk of catastrophic health expenditure or depth of impoverishment associated. Qualitative interviews were conducted to evaluate financial barriers and facilitators to care and were analyzed using thematic content analysis. Qualitative and quantitative data were triangulated to compare themes, identify areas of agreement or dissonance, and assess for complementarity.

**Results:**

Thirteen caregivers of children with cancer at KCMC formed the study cohort. Most lived in a rural setting (92%) and were farmers or livestock keepers (68%). Quantitative analysis showed that total median OOP health expenditures were $53.01 (IQR: 26.50–106.01). All families were pushed further into poverty from the OOP expenses as shown by widening poverty gaps. Qualitative interviews revealed several themes related to financial challenges for families with cancer, particularly during the time period prior to definitive care including worry about job losses and having to sell assets to reach care. Data triangulation confirmed strong agreement between qualitative and quantitative data on the impact of financial barriers on care. However, families stated higher OOP costs in qualitative interviews compared to quantitative data.

**Conclusions:**

Protecting families from impoverishment by reducing OOP costs during time periods prior to receiving definitive care may be a strategic way to improve timely diagnosis and early treatment for children with cancer.

## Introduction

Annually, over 500 million people are pushed into poverty or face catastrophic health expenditures due to out-of-pocket (OOP) health expenditures, particularly in low- and middle-income countries (LMICs) [1]. The risk of impoverishment due to OOP health expenditures for children is particularly high for conditions requiring lengthy and costly treatments such as cancer compared to other common childhood diseases [2,3].

Between 2020 and 2050, an estimated 11 million children will die from cancer, with 85% occurring in LMICs [4]. To improve these outcomes, the WHO Global Initiative for Childhood Cancer (GICC) has set a global goal of achieving at least 60% 5-year survival for children with cancer by 2030 through a comprehensive cancer care scale-up plan, including protection of families from cancer-related impoverishment [5]. Although the global health community recognizes the impact of OOP health expenditures on general health outcomes for children, the full extent of that risk remains unclear for children with cancer.

Tanzania is a lower-middle income country in Africa with a population of 62 million, of which 45% are children younger than 15 years of age [6]. Our previous research at the Kilimanjaro Christian Medical Center (KCMC) found a high incidence of childhood cancer, with 60% of children traveling from other regions [7,8].

Our study aims to estimate the impact of OOP health expenditures for cancer care for children at KCMC in Tanzania and to assess how OOP expenditures impact impoverishment through a mixed-methods approach. Our objectives were to estimate the OOP health expenditures for cancer care for the children prior to reaching KCMC, including medical and non-medical expenses, map the journey and financial expenditures from the time of first symptom recognition to reaching KCMC, and assess the impact of these expenditures on the family’s risk of impoverishment and catastrophic health expenditures. Understanding the impact of OOP expenditures may allow us to develop targeted interventions to protect children with cancer in LMICs from impoverishment and improve timely access to care.

## Methods

### Guiding framework

We used a convergent parallel mixed-method design based on the Three Delays Framework [9–11]. This model organizes delays in an individual’s health care in three stages: Delays in the decision to seek care (Delay 1), Delays in reaching definitive care (Delay 2), and Delays in receiving definitive care (Delay 3). We chose this model to specifically assess the financial risk of cancer care for families, as previous research has found that the costs of care differ depending on the stage in the care continuum [11,12].

### Overall study design and setting

Participants included caregivers of children with cancer selected by convenience sampling. We collected quantitative data from all participants using surveys to assess the risk of catastrophic health expenditures (CHE) and depth of impoverishment due to OOP health expenditures for the child’s cancer care. We collected qualitative data from interviews with the same participants to evaluate the financial barriers and facilitators of cancer care, with interviews analyzed using thematic content analyses and summarized across the Three Delay Framework. Quantitative and qualitative data were triangulated to identify areas of agreement, convergence, and clarification. We used the Consolidated Criteria for Reporting Qualitative Research (COREQ) guidelines for qualitative methods and the Consolidated Health Economic Evaluation Reporting Standards 2022 (CHEERS 2022) guidelines for quantitative methods. (S1 Appendix).

The study took place at KCMC, a tertiary zonal referral hospital in the Northern Region of Tanzania. KCMC is one of two pediatric and adult cancer centers in Northern Tanzania. KCMC hosts one of two pediatric oncologists in the northern Tanzania region, covering a catchment area of 15 million people. Financial coverage for direct medical expenses, including all conventional chemotherapy services and several targeted therapies as well as non-medical expenses such as food while is fully supported by KCMC, resulting in no costs to the families of children with cancer after initiating definitive care.

### Research team and reflexivity

The study team included a paid, bilingual, female Tanzanian research coordinator who is a medical doctor (HD) and conducted all interviews and is from the study area, KCMC’s pediatric oncologist (EM) who is from Tanzania and served as the co-PI of the study, two graduate students based in the United States (US) (MM, PE), a US-based mixed methods health researcher with 5 years of experience (AT), and an epidemiologist and co-PI with 10 years of global children’s health experience (ERS) from the US. Prior to interviews, the research coordinator (HD) was trained by AT in qualitative methods but had no interaction with participants prior to study commencement. It is acknowledged that the interpretation of the findings might be influenced by perspectives from the US-based team members. However, discussions and reflections with the Tanzanian co-PI (EM) and research coordinator (HD) provided insights into the local context and enriched the analysis with diverse perspectives and helped mitigate potential biases.

### Screening and enrollment

Study participants included caregivers of patients with cancer 0–14 years of age who were cared for at KCMC between June 30 and August 26, 2023. Families of children with cancer were screened for participation by the research coordinator (HD). Two families declined participation. Exclusion criteria included not speaking English or Swahili, or the inability to participate in interviews. If the patients’ families were eligible and interested in the study, the research coordinator (HD), who was fluent in Swahili and English, went through the informed consent process in a private location in the ward to obtain written informed consent, with a hardcopy of the informed consent form provided to the participant.

### Data collection

#### Quantitative data.

Participants first completed a quantitative financial risk assessment questionnaire initially developed by the Program in Global Surgery and Social Change at Harvard University (S2 Appendix) [13]. We adapted this tool to collect all direct medical and non-medical expenses spent on the child’s medical care for cancer from the time they started seeking care prior to the time they reached KCMC. Household expenses and all OOP expenses were estimated as the average monthly amount and included amounts spent on food/water, healthcare, transportation, other household goods, education fees, and livestock or farming activities. Direct medical expenses included amounts spent on hospital admissions fees, medications, laboratory tests, imaging and x-rays, treatments, medical supplies, and hospitalization. Non-medical expenses included amounts spent on childcare, food, lodging, additional needs, and amounts borrowed (if any). Participants were asked to provide the medical and non-medical estimates only related to the child’s cancer care from the time of first symptom recognition to receiving care at KCMC.

#### Qualitative data.

After completing the quantitative surveys, all participants underwent a semi-structured interview with research staff using a previously validated interview guide from the Harvard Medical School Program in Global Surgery and Social Change [13]. We adapted the guide to include additional questions about the financial impact of seeking care, including non-medical costs such as lodging and transportation, descriptions of any health facilities and/or traditional healers visited prior to KCMC, and to specify the time period where these expenses occurred. Participants were asked if the child’s cancer had other impacts such as lost wages or employment, but were not asked to quantify this impact. The interview guide was pre-evaluated for clarity and appropriateness by the research team (HD, EM, MM, ERS, CC-C), translated from English to Swahili, and back-translated by local healthcare professionals (HD, EM) fluent in both languages (S2 Appendix). The guide was pilot tested and refined with two caregivers of children who were receiving follow-up care at KCMC for hemophilia. Modifications to the interview guide after pilot testing were rephrasing of a few questions to be more linguistically and culturally appropriate.

Semi-structured interviews were conducted in a private location in the cancer ward by a local professional (HD) and lasted approximately 30–60 minutes. All interviews were conducted in Swahili, audio recorded for transcription, and then translated into English. All data were de-identified and participants were assigned a study unique ID.

### Outcome definitions and data analysis

#### Quantitative analysis.

We summarized the OOP expenses only related to delays in the decision to seek care (Delay 1) and delays in reaching definitive care (Delay 2), since expenses during the time period of receiving cancer care (Delay 3) were covered by KCMC. We classified healthcare seeking costs into two categories: medical and non-medical expenditures. The family’s income was summarized as the family’s average income per month in Tanzanian shillings (TZS) multiplied by the months the family sought care prior to reaching KCMC. All financial data were adjusted to international dollars using the purchasing power parity (PPP) conversion factor for Tanzania to correct for private consumption local currency unit per international dollar (754.6 for the year 2017) [14].

CHE was defined by the WHO definition as the total OOP costs faced for the child’s cancer care exceeding 10% of the household’s income during the months of seeking care [15]. Depth of impoverishment was defined as the household’s daily income minus the average daily OOP expenditure. Total OOP costs included all expenses paid out-of-pocket while seeking, reaching, or receiving care for the child’s cancer and included lodging, food, and transportation costs during this time. We also calculated the poverty gap at baseline (i.e., before Delay 1 of seeking care) and after reaching KCMC, defined as the mean shortfall income from the World Bank’s established extreme and standard poverty lines for low-income countries ($2.15 and $3.65, respectively) [16] for each family, controlled by household size [17].

#### Qualitative analysis.

A preliminary codebook of themes and sub-themes was deductively created before any interviews by the study principal investigator (PI) (ERS) based on the Three Delays model, followed by inductive thematic analysis after 10 interviews by the study PI (ERS) that generated additional codes (S3 Appendix). Interviews lasted under an hour and were performed in a quiet, private room in the KCMC pediatric oncology area using a semi-structured interview guide. Open-ended questions were aligned to the Three Delays Model. Interviews were recorded for transcription and translation, field notes were collected on paper and stored with the study files, and no repeat interviews were needed. Audio recordings, field notes, and study files were stored in a password-protected and encrypted repository only available to the qualitative team and paper files were stored in a locked cabinet at KCMC. Data was de-identified before electronically transferring to Duke University and stored in a password-protected, encrypted repository.

After 10 interviews, the study PI coded the interviews and organized findings using NVivo V.12 and 14 (Lumivero, Denver, CO, USA). To ensure data saturation, we conducted three more interviews with no new themes emerging. To ensure analytic rigor, inter-coder reliability was assessed through a random sample of four interviews (30%) were coded using the preset codebook by another set of reviewers (HD, PE), including the local team research coordinator (HD) [18,19]. Inter-coder reliability was high among all coders (Kappa = 0.91, 97–100% agreement). Method details can be found in S4 Appendix.

#### Data integration.

Data integration was performed through several approaches [20]. First, we compared the themes and sub-themes from the qualitative data with the quantitative data to assess if the financial barriers aligned with the quantitative data. Second, we compared the qualitative and quantitative data by theme to identify areas of agreement or dissonance. After the comparison, the study members came together to review the level of convergence. Third, we assessed if the qualitative data clarified or elaborated on themes from the quantitative data. Finally, we assessed how the qualitative data further expanded on any unsuspected quantitative results.

### Ethical considerations

This study was approved by the Kilimanjaro Christian Medical Centre Institutional Review Board (Protocol 2576), the Tanzania National Institute for Medical Research (NIMR: Protocol NIMR/HQ/R.8a/Vol.IX/4066), and the Duke University Institutional Review Board (Protocol 00110763). Patients or public were not involved in the study design and analysis.

## Results

Thirteen family members of children with cancer receiving treatment at KCMC formed the study cohort (**Table 1**). Most interviewees were the parent of the child (69%), lived in a rural setting (92%), did not have insurance (92%), and were farmers or livestock keepers (69%). The most common cancer types included eye cancers (31%) followed by muscle cancers (23%). The median monthly income for the families corrected to international dollars was 132.52 USD (IQR: 92.76–168.96), meaning all families were under the poverty line.

**Table 1:** Demographics characteristics of families who reached cancer care at KCMC.

	Total N (%) 13 (100.0)
**Interviewee’s Relationship to Child**	
Mother/Father	9 (69.2)
Other	4 (30.8)
**Sex**	
Female	6 (46.2)
Male	7 (53.8)
**Child’s age**	
0–6	7 (53.9)
7–14	6 (46.1)
**Child’s cancer type**	
Eye cancer	4 (31.0)
Kidney cancer	1 (7.7)
Leukemia	1 (7.7)
Muscle cancer	3 (23.0)
Unknown	4 (31.0)
**Household size**	
2–6	7 (53.8)
7+	6 (46.2)
**Rural/urban**	
Rural	12 (92.3)
Urban	1 (7.7)
**Occupation of main household provider**	
Farmer	6 (46.1)
Farmer and livestock keeper	1 (7.7)
Livestock farming	2 (15.4)
Self employed	3 (23.1)
Skilled employment	1 (7.7)
**Insurance**	
No	12 (92.0)
Yes	1 (7.7)
**Monthly household income** ^*^ **, median (IQR)**	
2017 PPP USD	132.52 (92.76 - 168.96)

IQR = interquartile range; PPP = purchasing power parity.

* Converted to USD and adjusted to 2017 PPP USD

### Quantitative results

**Fig 1:**
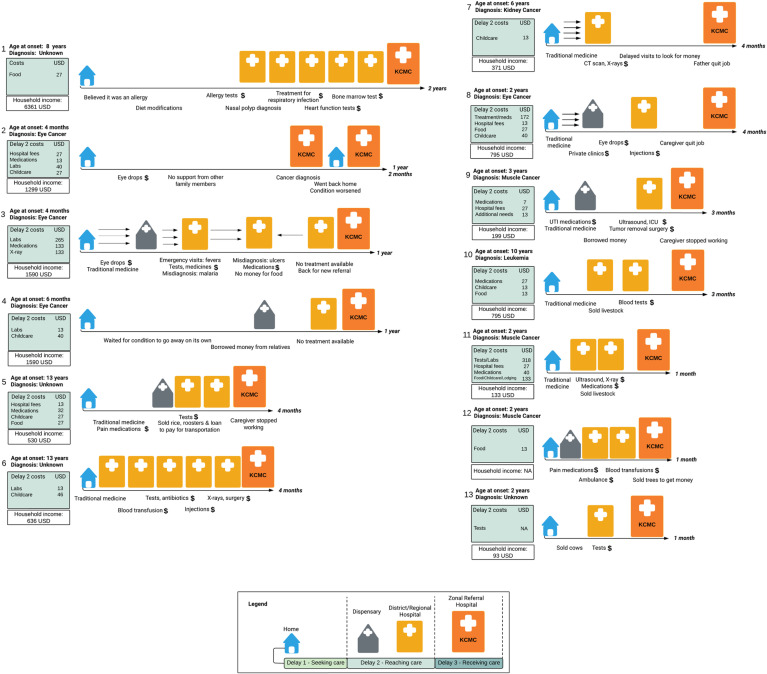
The journey of families to reach cancer care at KCMC. Note: Income and costs data converted to USD and adjusted to 2017 PPP. The monthly income for each family was multiplied by the number of months during which care was sought. For example, the income for family 3 was 132.5 USD per month and they sought care for 12 months, resulting in a total income of 1590 USD (2017 PPP) during the period this family was seeking care.

On average, the thirteen families spent 6.7 months seeking cancer care at an average of 3 other health facilities for their child during the Delay 2 period before reaching KCMC (**Fig 1**). For each family, we also included where the care was sought and any treatments or tests received before reaching KCMC based on the qualitative interviews. During that time, they spent 26.50 USD (IQR: 0.00–79.51) on direct medical expenses and 26.50 USD (IQR: 13.25–46.38) on non-medical expenses such as food, lodging, and transportation, for a total of 53.01 USD (IQR: 26.50–106.01) (**Table 2**).

**Table 2:** Impact of OOP expenditures on household finances and poverty gaps ($2.15 and $3.65 poverty lines) (n = 13).

FID	OOP Costs (in PPP Dollars)			Daily income before and after OOP expenses (in PPP Dollars)		Difference in poverty gaps after OOP expenses at $2.15 and $3.65 poverty lines			
	Medical costs	Non-medical costs	Total costs	Daily income per person	Daily income after OOP expenses per person	Poverty gap at baseline/after OOP expenses, $2.15 (%)	Difference in poverty gap after OOP expenses, $2.15 (%)	Poverty gap at baseline/after OOP expenses, $3.65 (%)	Difference in poverty gap after OOP expenses $3.65 (%)
1	0.00	26.50	26.50	1.26	1.26	41.30/41.54	0.59	65.42/65.57	0.22
2	79.51	26.50	106.01	0.39	0.35	82.02/83.49	1.79	89.41/90.28	0.97
3	530.07	0.00	530.07	0.74	0.49	65.76/77.17	17.36	79.83/86.55	8.42
4	13.25	39.76	53.01	0.88	0.85	58.91/60.28	2.33	75.80/76.60	1.06
5	45.06	53.01	98.06	0.74	0.60	65.76/72.09	9.63	79.83/83.56	4.67
6	13.25	46.38	59.63	0.88	0.80	58.91/62.76	6.54	75.80/78.07	2.99
7	0.00	13.25	13.25	1.03	0.99	52.06/53.77	3.29	71.76/72.77	1.41
8	185.52	66.26	251.78	0.95	0.63	55.97/70.65	26.22	74.07/82.71	11.67
9	33.13	13.25	46.38	0.32	0.24	85.32/88.75	4.01	91.36/93.37	2.21
10	26.50	26.50	53.01	0.68	0.41	68.39/81.04	18.49	81.38/88.83	9.15
11	384.30	132.52	516.82	0.74	−2.13	65.76/203.08	52.07	79.83/98.54	25.27
13	0.00	0.00	0.00	0.31	0.31	85.62/85.62	0.00	91.53/91.53	0.00
Median (IQR)	26.50 (0.00-79.51)	26.50 (13.25-46.38)	53.01 (26.5-106.0)	0.74 (0.61-0.90)	0.55 (0.34-0.81)	65.76 (58.17-71.80)/ 74.63 (62.14-84.02)	5.28 (2.19–17.64)	79.83 (75.36-83.39)/ 85.06 (77.70-90.59)	2.60 (1.04–8.60)

FID = Family ID; OOP = out-of-pocket; CHE = catastrophic health expenditure; IHE = impoverishing health expenditure; IQR = interquartile range; NA = not applicable; PPP = purchasing power parity.

Notes: Income converted to international dollars (adjusted to 2017 PPP). Family 12 was excluded due to missing income data.

**Fig 2:**
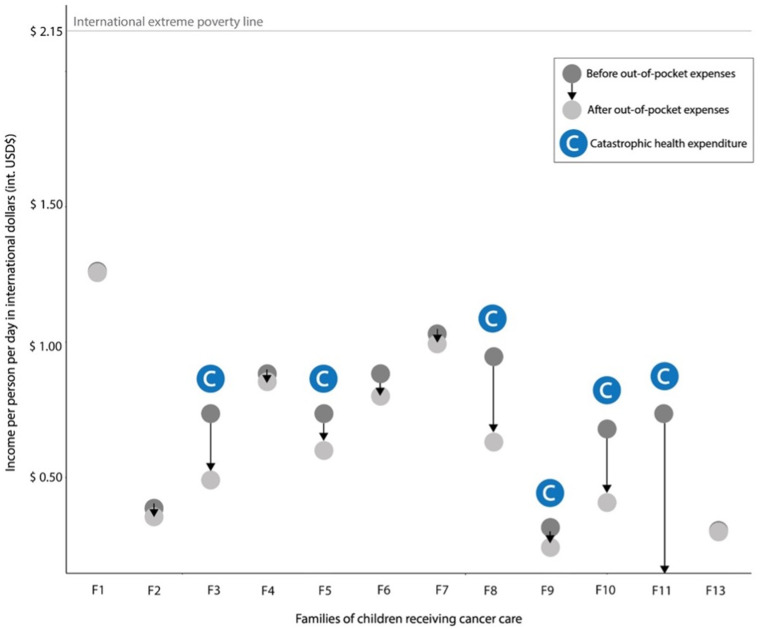
Impact of out-of-pocket expenses on families’ finances: catastrophic health expenditures and depth of impoverishment. Note: Family 12 was excluded due to missing income data. The arrow for family 11 goes to zero because this family had no money left after seeking care.

Daily income at baseline was 0.74 USD (IQR: 0.61–0.90) and was reduced to 0.55 USD (IQR: 0.34–0.81) after taking into account OOP expenses (**Table 2**). After accounting for OOP expenditures, the family’s daily income per person was reduced by a median of 0.19 USD, pushing the families further into poverty as shown by higher poverty gaps (**Fig 2**). The median poverty gap at baseline according to the $3.65 and $2.15 standard and extreme poverty lines, respectively, was 79.83 (IQR: 75.36–83.39) and 65.76 (IQR: 58.17–71.80). However, after OOP expenses were considered, the poverty gaps widened to 85.06 (IQR: 77.70–90.59) and 74.63 (IQR: 62.14–84.02).

### Qualitative results

Qualitative analysis of interview transcripts confirmed several themes regarding the facilitators and barriers to cancer care along the entire care continuum. In terms of the time period in the care continuum, most often cited themes were coded as Delay 1, mentioned 28 times in the interviews. See **Table 3** for thematic framework and example quotes.

**Table 3:** Key themes of seeking, reaching, and receiving care for children with cancer at KCMC (n = 13).

Three Delays Model	Sub-themes (number of citations from participants)	Quotes
Delay 1 – Seeking care	Lost job (n = 7)	*“I had to stop farming and leave my rice field to go with my child. I told the young ones to continue working on the farm, and I left to take care of my child. I will finish weeding the rice field when I come back …”* (F5)
	No money for food (n = 2)	“… *there were times when we didn’t have enough money even to buy food*.” (F3)
	Too far away (n = 1)	“*I used to have that worry about how I would pay for these costs. Coming from far away I was just hearing about Moshi never been here*…” (F8)
	Other children affected (n = 10)	“… we left our young children at home....When they feel like going [to school] it’s fine, but if they don’t feel like it, they don’t go.” (F11)
	Traditional healer (n = 4)	“*The first thing we consider is using traditional remedies to avoid the costs of hospitals. We fear the current hospital expenses.”* (F5)
	Worries about cost of medications/referrals (n = 4)	“*Sometimes, you receive a prescription, but you can’t afford the medicine. You go home with the prescription, and the patient dies because you don’t have the money … you use whatever resources you have … It all ends, and you find that nothing is left.”* (F1)
Delay 2 – Reaching care	Sold assets (n = 5)	“*We sold our livestock, cows, and goats, and got money to come here*.” (F11)
	Transport costs (n = 2)	“*So, I told him I couldn’t go to Moshi; I couldn’t afford to.*” (F7)
	Referral expenses high and led to delays (n = 8)	“*When you arrive at a hospital, they should give you a referral early because sometimes you arrive, and they perform tests and tests you find you have spent like 400,000 shillings and the child has not even improved. Later on, when your already broke that’s when they give you a referral*.” (F11)
Delay 3 – Receiving care	Lost job to keep child in care (n = 3)	*“I myself had to leave my job. I had started learning sewing, but I had to quit because of my child’s illness.”* (F9)
	Worries about paying for treatment (n = 2)	*“The main concern is the financial aspect, how to pay for the treatment.”* (F4)
	Financial facilitators (n = 3)	*“People are dying from this cancer, so let the service be provided for free.”* (F5)
	Financial worries after KCMC (n = 2)	*“… I have one worry: where will I get the fare to go back? Will KCMC support me again in returning until I reach my station? When I get home, I know I’ll have to rely on myself.”* (F5)

For Delay 1 ‘seeking care’, six sub-themes emerged. Worrying about other children being affected at home was the main barrier to seeking care for a child with cancer, stating that one would leave other children at home, which meant they would often miss school for several days a week. As well, participants worried about losing ones’ job if they sought care, the cost of medications or referrals, seeking care from a traditional healer since it is cheaper than the formal health system, not being able to afford food if they needed to pay for healthcare, and concerns about travel costs.

*“I had to stop farming and leave my rice field to go with my child. I told the young ones to continue working on the farm, and I left to take care of my child.” F5, Uncle, household size 6*

The predominant financial barrier for Delay 2 ‘reaching care’ was high expenses for tests at health facilities other than KCMC, followed by having to sell assets and high transportation costs to reach KCMC. The main financial barrier to Delay 3 ‘receiving care at KCMC was loss of the caregiver’s job due to needing to stay at the hospital with the child during treatment. Not related to any of the delays were worries of being able to afford the continuation of care when the child went home.

*“… I have one worry: where will I get the fare to go back? Will KCMC support me again in returning until I reach my station? When I get home, I know I’ll have to rely on myself.” F5, Uncle, household size 6*

The only facilitator emerged in Delay 3, which was not having to worry about treatment, food, or lodging while you were at KCMC. This facilitator seemed to ease the burden for many families.

*“If I go there and they give me an account statement saying I don’t owe anything and that it’s already paid for, then my worries will end right there.” F9, Mother, household size 7*

### Data triangulation

We found strong agreement between the qualitative data and quantitative data for Delay 1 and 2 barriers. The main barriers identified in the qualitative data included having to pay for medications or tests during the referral period, costs for transportation and lodging, and losing one’s job due to taking care of the child’s cancer. The quantitative data supported the quantitative findings by clearly identifying which OOP expenses were incurred during Delays 1 and 2. There was some disagreement between the datasets seen by complementarity analysis. In three interviews, the family mentioned expenses during the Delay 2 period that were not included in the quantitative data collection. For example, although one family mentioned spending 400,000 Tanzanian schillings ($146.80 USD) on referrals and tests prior to the child’s diagnosis during the interview, the total OOP costs were reported lower ($80.00 USD) in the quantitative data collection. The other two families mentioned slightly higher OOP costs in the qualitative interviews than were reported in the quantitative data, suggesting a potential bias in the final OOP cost estimates.

## Discussion

Results from our study show that financial barriers are particularly challenging for the time period prior to the child receiving definitive cancer treatment. These findings highlight the importance of lowering OOP costs during the care-seeking period for a child with cancer in LMICs. Additionally, the families in our study had long delays receiving care, indicating the need to strengthen early diagnosis and referral networks.

Our study confirmed previous findings that high OOP costs can have a significant impact on families with a child with cancer in LMICs [11,12,21,22]. A systematic review reported that cancer patients living in LMICs spend about 42% of their annual income on OOP expenses, compared to 16% in high income countries [23]. Higher OOP costs have also been observed among pediatric cancer patients compared to adult cancer patients [2,3]. Of note, these previous studies only assessed OOP costs during the time of receiving cancer care, not prior to initiating care. Our study fills in the gap by demonstrating high OOP expenditures during the care seeking time may be just as impactful than after treatment was initiated.

The impact of universal health coverage (UHC) to provide access to health services may be limited, especially among the poorest populations [24]. Although UHC is intended to provide free necessary healthcare to populations that need it the most, our data shows that current UHC schemes may not be enough to protect families of a child with cancer from impoverishment due to OOP expenses, particularly prior to receiving health services which are mainly non-medical. One strategy to reduce the financial burden on families of a child with cancer is through alternative financial protection mechanisms, including cash transfer programs such as the Bolsa Familia Project in Brazil. These programs are associated with reducing child mortality with the greatest impact among families in poverty [25]. Another strategy may be to specifically target financial protection mechanisms for uninsured families, knowing that OOP health expenditures are higher for families who are uninsured in LMICs [26]. Expanding our definition of financial protection mechanisms from UHC to all OOP expenses could strategically and effectively protect families from impoverishment.

Timely treatment for childhood cancer is dependent on timely diagnosis and strong referral networks along with protection against poverty. Our data shows that families of a child with cancer in rural areas often experience a double burden, with underlying high rates of poverty as well as lengthy journeys to receive definitive care. However, strong referral networks can improve a child’s health outcomes. For example, Rwanda has built a strong referral network and infrastructure for children with cancer by linking local physicians with remote specialist consultants and improving diagnostic and treatment capacity in rural districts [27]. Our data augments these findings to suggest that strengthening the referral network may reduce OOP expenses for families and link families to definitive care earlier.

There are several limitations to our study. First, our study was conducted in a single institution with a relatively small sample size, limiting the generalizability of study findings. The objective of the study was to assess the financial impact of a child’s cancer care on families through mixed-methods and was not powered for quantitative analyses. Although our sample size was small for quantitative purposes, the qualitative interviews among the 13 families reached saturation. Future studies with larger sample sizes in multiple institutions would increase our ability to draw significant quantitative conclusions although that was not the primary purpose of the current study. Second, our interviews asked participants about their OOP expenses during the care-seeking time prior to coming to KCMC and the average monthly income, raising the risk of recall bias. Although there is a potential for inaccuracies in OOP and income measurements due to recall bias, we were able to triangulate the quantitative responses with the qualitative responses and found strong agreement except for two families (discussed next). Third, the qualitative interviews for two families showed that the financial burden to be different than what was collected in the quantitative data. After discussing this finding with the study team during the triangulation process, the main reason for this discrepancy appears to be the difficulty to accurately quantify how much each family spent for OOP expenditures. However, the interviews may be a better avenue to elucidate OOP expenditures, as the family member may have felt more comfortable sharing their story rather than estimating a distinct number. Lastly, we did not include indirect costs resulting from seeking or receiving healthcare, such as lost wages to the family. Although the interviews did not capture when the caregivers mentioned their jobs were disrupted or lost due to taking the child to the various health appointments, our quantitative data collection sheet did not quantify this loss.

In conclusion, our study identified several financial challenges during the period before reaching care for children with cancer and highlights the high risks of impoverishment related to non-medical OOP costs during this time period. Our data suggests that protecting families from impoverishment by reducing OOP costs may be a strategic way to ensure children obtain timely treatment for cancer and may be critical to advance to the GICC goal of reducing childhood cancer mortality to 60% by 2030.

## Supporting information

S1 Appendix Checklists. (DOCX)

S2 Appendix Data collection semi-structured interview guide and quantitative data collection sheet. (DOCX)

S3 Appendix Codebook and themes/sub-themes. (DOCX)

S4 Appendix Method details. (DOCX)

## References

[1] World Health Organization. Global monitoring report on financial protection in health 2019. Geneva, Switzerland; 2019. https://www.who.int/healthinfo/universal_health_coverage/report/fp_gmr_2019.pdf?ua=1

[2] Chae W, Kim J, Park S, Park E-C, Jang S-I. The financial burden associated with medical costs among childhood cancer patients and their families related to their socioeconomic status: the perspective of national health insurance service. Int J Environ Res Public Health. 2020;17(17):6020. doi: 10.3390/ijerph17176020 32824940 PMC7503756

[3] Anhang Price RR, Stranges ETR, Elixhauser AA. Pediatric Cancer Hospitalizations, 2009. HCUP Statistical Brief #132. Rockville, MD: Agency for Healthcare Research and Quality; 2012. http://www.hcup-us.ahrq.gov/reports/statbriefs/sb132.pdf. 22787680

[4] Organization WH. Childhood Cancer. 2020. Available from: https://www.who.int/news-room/fact-sheets/detail/cancer-in-children.

[5] World Health Organization. Global Initiative for Childhood Cancer. [cited 2023 Jun 15]. 2021. Available from: https://www.who.int/cancer/childhood-cancer/en/

[6] Administrative Units Population Distribution Report. [cited 2024 Aug]. Available from: https://www.nbs.go.tz/nbs/takwimu/Census2022/Administrative_units_Population_Distribution_Report_Tanzania_volume1a.pdf

[7] Majaliwa E, Smith ER, Cotache-Condor C, Rice H, Gwanika Y, Canick J, et al. Childhood and Adolescent Cancer Care at a Tertiary Hospital in Northern Tanzania: a retrospective study. JCO Glob Oncol. 2023;9:e2200263. doi: 10.1200/GO.22.00263 37384861 PMC10497254

[8] Gwanika Y, Rice HE, Metcalf M, Espinoza P, Kajoka HD, Rice HE, et al. Impact of the COVID-19 pandemic in childhood and adolescent cancer care in northern Tanzania: a cross-sectional study. BMC Cancer. 2024;24(1):457. doi: 10.1186/s12885-024-12168-y 38609910 PMC11010397

[9] Thaddeus S, Maine D. Too far to walk: maternal mortality in context. Soc Sci Med. 1994;38(8):1091–110. doi: 10.1016/0277-9536(94)90226-7 8042057

[10] Concepcion TL, Dahir S, Mohamed M, Hiltbrunn K, Ismail EA, Poenaru D. Barriers to surgical care among children in Somaliland: an application of the three delays framework. World J Surg. 2020:1–7. 10.1007/s00268-020-05414-4 32030443

[11] Cotache-Condor C, Kantety V, Grimm A, Williamson J, Landrum KR, Schroeder K. Determinants of delayed childhood cancer care in low- and middle-income countries: a systematic review. Pediatr Blood Cancer. 2023;70(3):e30175. 10.1002/pbc.30175 PMC10966942 36579761

[12] Cotache-Condor C, Rice HE, Schroeder K, Staton C, Majaliwa E, Tang S, et al. Delays in cancer care for children in low-income and middle-income countries: development of a composite vulnerability index. Lancet Glob Health. 2023;11(4):e505–15. doi: 10.1016/S2214-109X(23)00053-0 36925171 PMC10938288

[13] Program in Global Surgery and Social Change. National Surgical, Obstetric and Anaesthesia Planning (NSOAP) Surgical Indicators. Financial Risk Protection Survey. 2021 Available from: https://www.pgssc.org/_files/ugd/d9a674_8da150554fe348f4bfcba71613faad9d.pdf

[14] World Bank. PPP conversion factor, private consumption (LCU per international $) – Tanzania. 2023. [cited 2023 Nov 3]. Available from: https://data.worldbank.org/indicator/PA.NUS.PRVT.PP?locations=TZ

[15] World Health Organization & International Bank for Reconstruction and Development. Global monitoring report on financial protection in health 2021. 2021. World Health Organization. https://iris.who.int/handle/10665/350240

[16] World Bank. Fact Sheet: An Adjustment to Global Poverty Lines. 2020 Available from: https://www.worldbank.org/en/news/factsheet/2022/05/02/fact-sheet-an-adjustment-to-global-poverty-lines#1

[17] World Bank. Fact Sheet: An Adjustment to Global Poverty Lines. 2020 [cited 2023 Mar 17]. Available from: https://www.worldbank.org/en/news/factsheet/2022/05/02/fact-sheet-an-adjustment-to-global-poverty-lines#1.

[18] Morse JM. critical analysis of strategies for determining rigor in qualitative inquiry. Qual Health Res. 2015;25(9):1212–22. doi: 10.1177/1049732315588501 26184336

[19] McHugh ML. Interrater reliability: the kappa statistic. Biochem Med (Zagreb). 2012;22(3):276–82. 23092060 PMC3900052

[20] Farmer T, Robinson K, Elliott SJ, Eyles J. Developing and implementing a triangulation protocol for qualitative health research. Qual Health Res. 2006;16(3):377–94. doi: 10.1177/1049732305285708 16449687

[21] Smith ERCT, Mohamed M, Dahir S, Adan E, Rice HE. Modeling the limitations of reducing out-of-pocket costs for children’s surgical care in Somaliland: the poorest of the poor need more. BMJ Open. 2023. 10.1136/bmjopen-2022-069572 PMC10163539 37130683

[22] Smith ER, Kapoor P, Concepcion T, Ramirez T, Mohamed M, Dahir S, et al. Does reducing out-of-pocket costs for children’s surgical care protect families from poverty in Somaliland? A cross-sectional, national, economic evaluation modelling study. BMJ Open. 2023;13(5):e069572. doi: 10.1136/bmjopen-2022-069572 37130683 PMC10163539

[23] Iragorri N, de Oliveira C, Fitzgerald N, Essue B. The out-of-pocket cost burden of cancer care-a systematic literature review. Curr Oncol. 2021;28(2):1216–48. doi: 10.3390/curroncol28020117 33804288 PMC8025828

[24] WHO. Health systems financing: the path to universal coverage. Geneva: WHO; 2010. 10.2471/BLT.10.078741 PMC2878164 20539847

[25] Ramos D, da Silva NB, Ichihara MY, Fiaccone RL, Almeida D, Sena S, et al. Conditional cash transfer program and child mortality: a cross-sectional analysis nested within the 100 Million Brazilian Cohort. PLoS Med. 2021;18(9):e1003509. doi: 10.1371/journal.pmed.1003509 34582433 PMC8478244

[26] Kagaigai A, Anaeli A, Grepperud S, Mori AT. Healthcare utilization and catastrophic health expenditure in rural Tanzania: does voluntary health insurance matter? BMC Public Health. 2023;23(1):1567. doi: 10.1186/s12889-023-16509-7 37592242 PMC10436390

[27] Stulac S, Mark Munyaneza RB, Chai J, Bigirimana JB, Nyishime M, Tapela N, et al. Initiating childhood cancer treatment in rural Rwanda: a partnership-based approach. Pediatr Blood Cancer. 2016;63(5):813–7. doi: 10.1002/pbc.25903 26785111

