# Supplementary material for: Impact of out-of-pocket expenses on children with cancer in Tanzania: A mixed-methods economic study
Source: PLoS One. 2025 Jun 26;20(6):e0326755. doi: 10.1371/journal.pone.0326755 (PMC12200705; doi:10.1371/journal.pone.0326755)
Supplement: S2 Appendix — (DOCX) [file pone.0326755.s002.docx]

**Appendix 2. Data collection semi-structured interview guide and quantitative data collection sheet**

**SEMI-STRUCTURED INTERVIEW GUIDE**

**Framing Statement:** The purpose of this interview is to ask you a few questions about your journey here to obtain care for your child. We would like to understand the challenges you face as well as the main areas for improvement here.

**Verbal Consent:** Do I have your permission to continue with this interview? Do you have any questions before we get started? **Note: Complete informed consent document before starting recording

**Participant Information:** **Note: All families should complete the quantitative guide in addition to this interview. Make sure the participant ID matches on both documents.

| **Name of Interviewer** |  |
| --- | --- |
| **Participant ID Number** |  |
| **Date of interview (dd/mm/yyyy)** | **\| ___ \| ___ \| ___ \| ___ \| ___ \| ___ \| ___ \| ___ \|** |
| **Location of interview** |  |
| **Relationship to patient** |  |
| **Participant’s gender** |  |
| **Participant agrees the interview to be electronically recorded** | - **Yes** - **No** |
| **Time interview starts** | **\| ___ \| ___ \|: \| ___ \| ___ \| am/pm** |
| **Time interview ends** | **\| ___ \| ___ \|: \| ___ \| ___ \| am/pm** |

[START RECORDING]

**Opening Statement:** We are here to learn from you and your experience and to identify how to improve cancer care for children in the future. I would like to remind you that there is no right or wrong answer in the questions that I am going to ask you. We want to hear your perspective. You can stop the interview at any point or skip any questions you’re uncomfortable answering.

**Opening Question:**

1. Can you tell me what illness your child is currently seeking treatment for?

**Barriers and Enablers in Seeking Care:**

1. What the reasons that made you seek care for your child? (symptoms/initial signs of illness)
2. How long did it take you to seek care from when you first noticed the symptoms? (exact time/days)
   1. **Probe (if delayed seeking care):** What were the reasons that made you delay seeking care?
3. Did you seek care from any traditional healers before arriving here?
   1. **If yes:** What were your motivations for seeking care through a traditional healer (economic, spiritual, convenience/geographic proximity, etc)?
   2. **If no:** What were the reasons you didn’t?

**Barriers and Enablers in Reaching Care:**

1. About how long has your child been at this facility since they have been diagnosed? (overall)
2. Can you tell me more about the journey from your home to this current facility?
   1. **Probe:** Did you seek care at any hospitals/clinics before this one?
      1. **If yes:** Where? How many?
      2. **If yes:** What kind of treatment/care did the child receive?
      3. **If yes:** How do you feel about the referral system to reach care in this region?
3. Did you face any challenges that prevented you from seeking care earlier for your child?
   1. **If yes:** What did you have to do to get your child to the hospital?
4. About how much time did you spend traveling to this facility?
   1. **Probe:** What was your experience like traveling with your ill child?
5. Do you have any worries about being away from home while your child obtains care?
   1. **If yes:** What are these worries?
   2. **If no:** What helps you feel comfortable being away from home?

**Barriers and Enablers in Receiving Care:**

1. What treatment is your child getting here at KCMC?
   1. **Probe:** Has there been any challenges specifically in obtaining this treatment for your child?
      1. **If yes:** Can you tell us about those challenges?
   2. **Probe:** How long do you expect the full course of treatment to take?
2. Do you feel confident that your child will complete his/her course of treatment at this facility?
   1. **If yes:** What makes you hopeful that your child can complete the treatment?
   2. **If no:** What barriers or fears do you face in completing your child’s treatment?

**Financing Care:**

1. Do you feel any financial stress about how you will pay for medical expenses?
   1. **If yes:** Can you expand upon this worry?
      1. **Probe:** What resources may allow you to feel less concerned about this?
   2. **If no:** What has enabled you to not feel concerned about this?
2. Has anyone in your household lost wages or employment due to seeking care for your child’s diagnosis?
   1. **If yes:** How will this impact your family life?

**Closing Questions:**

1. What suggestions do you have to improve the diagnosis and treatment for your child?
2. Thank you for taking the time to talk with me today, your perspective is very valuable. Is there anything else that may help us understand the challenges you have faced?

[END RECORDING]

**QUANTITATIVE DATA COLLECTION SURVEY**

**Framing statement:** The purpose of this interview is to ask you a few questions about your journey here to obtain care for your child. We would like to understand the challenges you face as well as the main areas for improvement here.

**Participant Information:**

Participant ID: ___________________ Date (mm/dd/yyyy): _______________

Interviewer Name: ____________________________ Hospital: __________________

**Household Demographics**

1. What is your relationship with the child? ___________________________
2. What is the child’s age? Years ________ Months (if years = 0) __________
3. What is the child’s sex? __________________
4. What is the classification of the child’s condition or illness? (surgery/cancer) __________
5. How many family members live in the household?

# of adults _______ # of children _______

1. How would you describe your home city? (urban/rural) ___________________
2. What ethnic group and/or tribe do you most identify with? (circle one)

Masaai Chagga Pare Sambaa Sukuma Mmeru

Nyaturu Muha Iraq None Other (specify): __________

1. What religious affiliation do you most currently identify with? (circle one)

Muslim Christian None Other (specify): __________

1. What occupation is held by the main household provider? (circle one)

Farmer Skilled Employment Professional Self Employed

1. What is your education level? (circle one)

None Primary Secondary College Postgraduate

1. Do you have any kind of insurance or health coverage? (Yes/No) ___________

| **Monthly Household Finances** | |
| --- | --- |
| Average monthly income |  |
| Amount spent on food/water each month |  |
| Amount spent on healthcare each month |  |
| Amount spent on transportation each month |  |
| Amount spent on other household goods each month |  |
| Amount spent on education each month |  |
| Amount spent on livestock or farming activities each month |  |

| **Direct Medical Expenses** | | **Non-Medical Expenses** | |
| --- | --- | --- | --- |
| Amount spent on hospital admissions fees |  | Amount spent on childcare |  |
| Amount spent on medications |  | Amount spent on food |  |
| Amount spent on laboratory tests |  | Amount spent on lodging |  |
| Amount spent on imaging and x-rays |  | Amount spent on additional needs |  |
| Amount spent on treatment |  | Amount borrowed (if any) |  |
| Amount spent on hospitalization |  | **TOTAL DIRECT EXPENSES** |  |
| Amount spent on medical supplies |  | **TOTAL NON-MEDICAL EXPENSES** |  |
