# Supplementary figures and images for: Impact of out-of-pocket expenses on children with cancer in Tanzania: A mixed-methods economic study
Source: PLoS One. 2025 Jun 26;20(6):e0326755. doi: 10.1371/journal.pone.0326755 (PMC12200705; doi:10.1371/journal.pone.0326755)

**Appendix 3. Codebook themes and sub-themes**


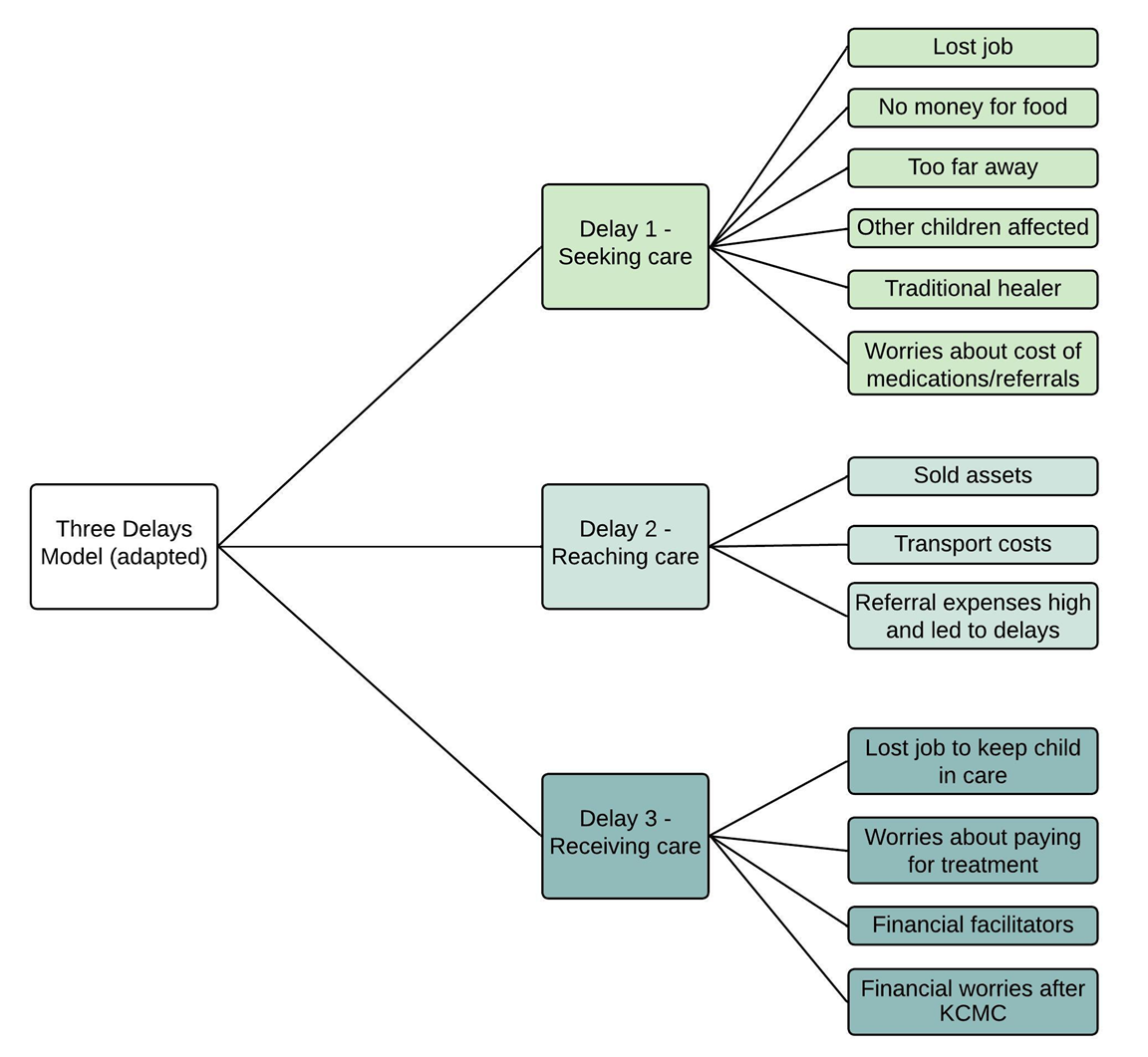

Supplement: S3 Appendix — (DOCX) [file pone.0326755.s003.docx]
