# Supplementary material for: Impact of out-of-pocket expenses on children with cancer in Tanzania: A mixed-methods economic study
Source: PLoS One. 2025 Jun 26;20(6):e0326755. doi: 10.1371/journal.pone.0326755 (PMC12200705; doi:10.1371/journal.pone.0326755)
Supplement: S4 Appendix — (DOCX) [file pone.0326755.s004.docx]

**Appendix 4. Method details**

**Study Team and Reflexivity**

The study team included a paid, bilingual, female research coordinator who is a medical doctor (HD) and conducted all interviews and is from the study area, KCMC’s pediatric oncologist (EM) who also served as the co-PI of the study, two graduate students (MM, PE), a mixed methods health researcher with 5 years of experience (AT), and an epidemiologist and co-PI with 10 years of global children’s health experience (ERS). Prior to interviews, the research coordinator (HD) was trained by AT in qualitative methods but had no interaction with participants prior to study commencement.

**Quantitative methods**

Direct medical expenditures included payments made for surgery, medical/diagnostic imaging, laboratory, medicines, traditional healers, and other medical supplies. Direct non-medical expenditures included payments made for transport to the hospital, hire of hospital attendants, and food expenses during hospitalization. Quantitative outcome definitions are expressed as follows:

**Poverty gap**

At the national level, the World Bank defines the poverty gap as the mean shortfall in income from the poverty line ($2.15 or $3.65). We calculated the poverty gap for each family member before and after care was sought using the formula below:

1. Poverty gap:

$$PG \left( \% \right) = \frac{l-i_{p}}{l}x 100$$

Where, *l* is the poverty line at either $2.15 or $3.65 per person per day and 𝑖𝑝 is the share of the household’s income for each family member per day.
